# Cerebral abscesses with odontogenic origin: a population-based cohort study

**DOI:** 10.1007/s00784-023-04976-6

**Published:** 2023-03-31

**Authors:** Frederik V. B. Jespersen, Signe U.-B. Hansen, Simon S. Jensen, Lars H. Omland, Jannik Helweg-Larsen, Thomas Bjarnsholt, Claus H. Nielsen, Morten Ziebell, Jacob Bodilsen, Merete Markvart

**Affiliations:** 1 Cariology and Endodontics, Section of Clinical Oral Microbiology, Department of Odontology, University of Copenhagen, Nørre Allé 20, 2200 Copenhagen, Denmark; 2 Department of Oral & Maxillofacial Surgery, Copenhagen University Hospital, Copenhagen, Denmark; 3 Department of Infectious Diseases, Copenhagen University Hospital, Copenhagen, Denmark; 4 Department of Clinical Microbiology, Copenhagen University Hospital, Copenhagen, Denmark; 5 Department of Immunology and Microbiology, University of Copenhagen, Copenhagen, Denmark; 6 Institute for Inflammation Research, Department of Rheumatology and Spine Disease, Copenhagen University Hospital, Copenhagen, Denmark; 7 Department of Neurosurgery, Copenhagen University Hospital, Copenhagen, Denmark; 8 Department of Infectious Diseases, Aalborg University Hospital, Aalborg, Denmark

**Keywords:** Brain abscess, Dental infection control, Microbiology, Oral health, Oral pathology, Panoramic radiography

## Abstract

**Objectives:**

Recent studies have indicated that cerebral abscess (CA) patients with odontogenic origin are on the rise. However, CA patients are often poorly characterized and with an unknown etiologic background. The purpose of this study is to identify and characterize CA patients that may have an odontogenic origin based on microbiologic, radiographic, and/or clinical findings.

**Materials and methods:**

This is a population-based cohort study analyzing retrospective and prospective data from CA patients. Radiographic examinations of panoramic radiographs (PRs) or computed tomography (CT) scans were conducted. CA patients characterized with odontogenic origin required the fulfilment of the following criteria on admission: (1) Oral pathologic conditions were the only bacterial infections present, (2) oral microorganisms were isolated in the purulent exudate from the brain, and (3) radiographically and/or clinical recordings of oral pathologic conditions.

**Results:**

A total of 44 patients could be included in this study of which 25 (57%) were characterized as having CA with a likely odontogenic origin. Type two diabetes (T2D) (*p* = 0.014) and microorganisms of the *Streptococcus anginosus* group (SAG) (*p* < 0.01) were overrepresented in patients with CAs of odontogenic origin.

**Conclusions:**

Odontogenic infections may cause CAs to a greater extent than previously assumed. T2D was overrepresented among patients with odontogenic CA. When microorganisms of the SAG were isolated from the brain pus, CA patients had a predisposing odontogenic or sinus infection.

**Clinical relevance:**

The identification of patients with a likely odontogenic CA will contribute to understanding the etiology of the infectious disease and highlighting the importance of preserving oral health.

**Supplementary information:**

The online version contains supplementary material available at 10.1007/s00784-023-04976-6.

## Introduction

Cerebral abscesses (CAs) are often associated with high morbidity and mortality, and in a large proportion of CA patients, the origin of the infection is unknown [1, 2]. Identifying the origin is crucial in preventing and treating the infectious disease, as elimination of the primary focus of infection should be a priority to avoid the spread of microorganisms [3, 4]. From 1982 to 2016, an increasing incidence of CAs has been observed in Denmark [5], and the number of patients with unknown source of infection has grown [1]. Consistent reports of oral microorganisms isolated from CA lesions have made odontogenic infections widely recognized as a potential source of infection [5]. Still, a meta-analysis including studies from 1970 to 2013 identified odontogenic predisposing infections in only 5% of the patients [3]. However, the included studies showed great heterogeneity precluding further analysis of the predisposing infections described. In recent studies, CAs of odontogenic origin have been reported to be more frequent [5, 6]. The reasons for this may be that more teeth are preserved nowadays [6], and there is an increased awareness that oral infections may spread to distant anatomical sites. Because of the increasing incidence of odontogenic CAs, a comprehensive understanding of their pathogenesis has become increasingly important. However, only a few studies have focused on odontogenic CAs. The purpose of the present study is to identify and characterize CAs of odontogenic origin in a well-defined cohort based on clinical, radiographic, and microbiologic findings.

## Methods

### Setting

The study was conducted as a retrospective population-based cohort study identifying all CA patients treated at the Department of Infectious Diseases (ID), Copenhagen University Hospital, Rigshospitalet (RH), from January 2015 to January 2019. Permission to perform the study and to handle the data was granted by the Danish Data Protection Agency (record no. 3–3013-2579/1) and the Regional Scientific Ethical Committees of The Capital Region of Denmark (record no. H-20046532).

### Data source

All patients diagnosed with first-time CA were identified through the Danish Study Group of Infections of the Brain (DASGIB) database, as described in detail previously [7]. In brief, DASGIB is a nationwide Danish observational prospective database in which all patients with infections located in the central nervous system are documented prospectively. Data used from the DASGIB database were registered by the local DASGIB representative or by specially trained research assistants during hospitalization [7]. All data were registered in a standardized web-based case report form using Research Electronic Data Capture (REDCap, Tennessee, TN, USA) [8]. Additional information was collected retrospectively by the principal investigator reviewing electronic medical records for all patients.

### Data extraction

From the DASGIB database, study variables including patient demographics, duration of hospitalization, predisposing infections, comorbidities, immunocompromising factors, predisposing risk factors, surgical interventions, microbiological findings, antibiotic treatment, and neuroimaging descriptions were collected. Data from dental examinations during hospitalization including observations from the oral maxillofacial surgeon and oral radiographic material were collected retrospectively from the medical records.

### Inclusion and exclusion

**Fig. 1:**
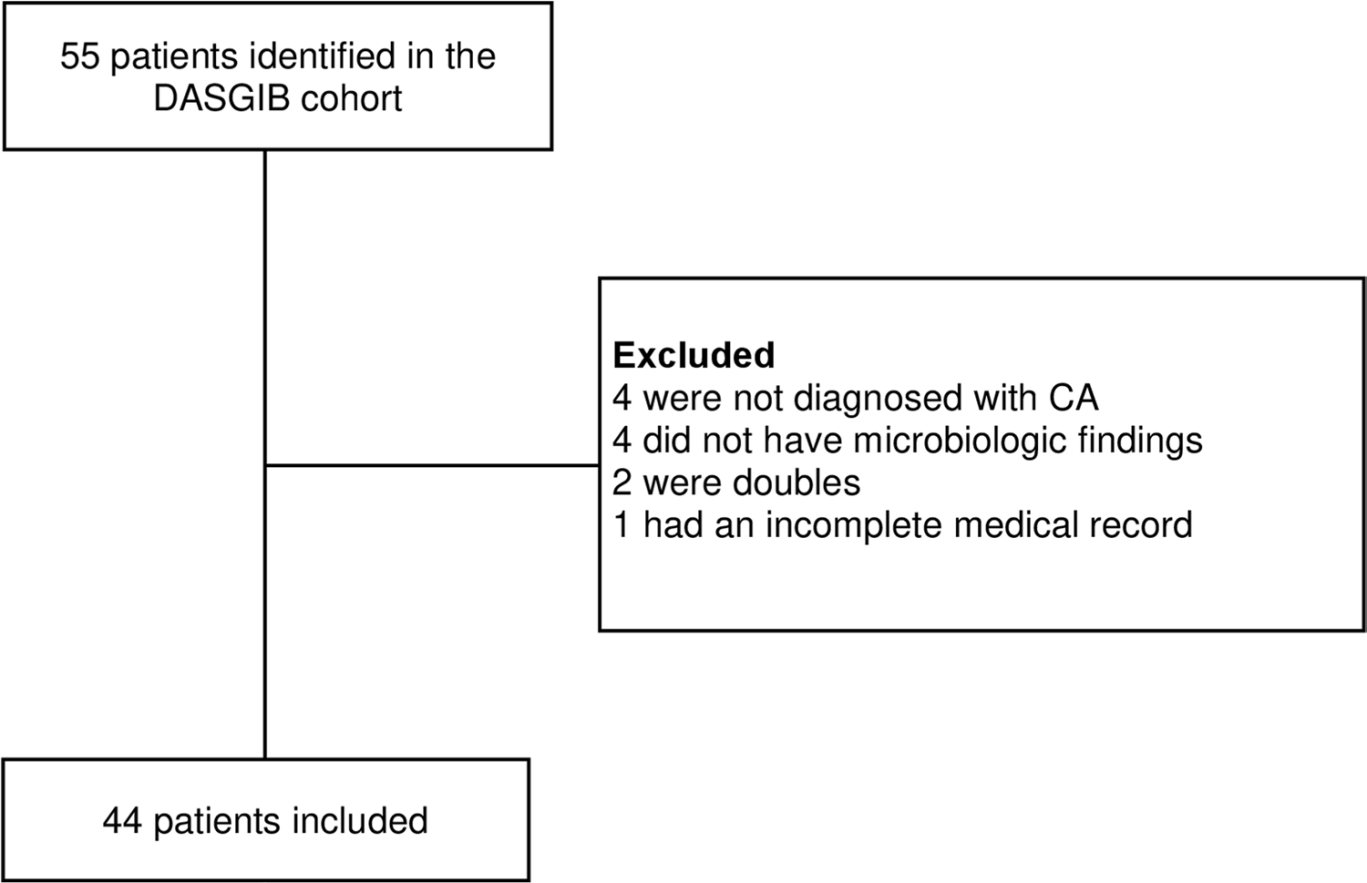
Flow of patients. Patients with CA included in the DASGIB cohort were all screened for eligibility. Patients were excluded if they did not meet the inclusion criteria. In total, 44 patients were included

Patients included in this study were diagnosed with CA based on neuroimaging described by a radiologist and verified by the identification of microorganisms in purulent exudate from the brain. Patients with no microorganisms isolated, lack of neuroimaging data compatible with CA, incomplete medical records, or doubles were excluded. The flow of patients is illustrated in Fig. 1.

### Radiographic assessment

Digital panoramic radiographs (PRs) were taken during hospitalization at the Department of Diagnostic Radiology, RH (Instrumentarium Dental, OP200D, Tuusula, Finland). All available PRs were characterized and evaluated twice, 1 month apart by four independent examiners (FVBJ, SUBH, SSJ, and MM) to evaluate intra- and inter-rater reliability. Kappa values (*κ*-values) were computed for six radiographic variables: the presence of endodontically treated teeth, retained teeth, signs of oral pathologic conditions like deep carious lesions, periapical radiolucencies, severe marginal bone loss, and pericoronal radiolucencies (Supplementary S1). For inter-rater reliability, all examiners had *κ*-values computed for the six variables, then the arithmetic means of these estimates were used to provide an overall index of agreement for each variable [9] (Supplementary S2). All *κ*-values were interpreted as previously suggested [10]. In a limited number of cases where PRs were not available, computed tomography (CT) scans of the maxillary and mandibular region were evaluated in cooperation, and agreement was achieved among the examiners.

### Characterization of CA with odontogenic origin

The patients were characterized as having either odontogenic or non-odontogenic CA based on criteria previously proposed [11]. Patients with CAs characterized as being of odontogenic origin required the fulfilment of the three following criteria:

1. Oral pathologic conditions were the only bacterial infections present on admission. Alternative bacterial infections were ruled out based on the anamnesis, clinical data, diagnostic imaging as described by the radiologist, and doctor’s notes from the medical records. It is a part of the standard procedure that the focus of infection is searched for in all CA patients by an infectious disease specialist during hospitalization.
2. Microorganisms identified in the purulent exudate from the brain were compatible with microorganisms that are part of the oral microbiota, involved in dental infections, and registered in the Human Oral Microbiome Database (HOMD).
3. Radiographically and/or clinical recordings of existing oral pathologic conditions including apical periodontitis, pericoronitis, and/or severe periodontitis.

### Sample processing

Samples for microbiological analyses were collected by neurosurgical aspiration or abscess evacuation. Both anaerobic and aerobic culturing on appropriate growth media as well as 16 s sequencing were performed following standard operating procedures at the hospital.

### Statistical analyses

All statistical analyses were computed using SPSS Statistics version 28.0 (IBM Corporation, Armonk, NY). Categorical variables were evaluated using the two-tailed Fisher’s exact test, and continuous variables were analyzed using the Student’s *t-*test. For continuous variables, results were presented as median or mean ± standard deviation (SD). To estimate the intra-rater and inter-rater reliability, Cohen’s kappa and Light’s kappa were applied respectively. The level of significance was set at *p* ≤ 0.05.

## Results

### Clinical characteristics

**Fig. 2:**
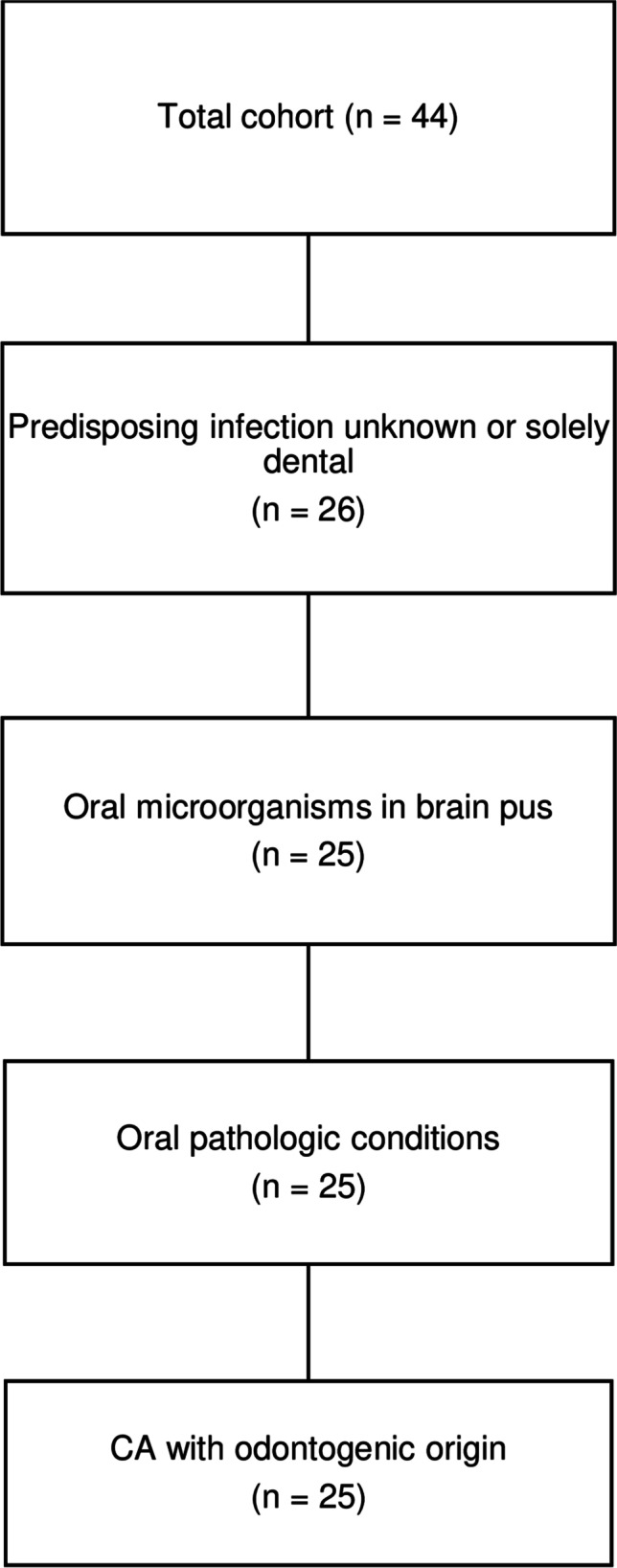
Flowchart showing the characterization of CA patients with odontogenic origin

Between 2015 and 2019, 55 patients with first-time CA were hospitalized at the ID department, RH. A total of 44 patients were included for further analyses (Fig. 1). Twenty-five patients (57%) were characterized as having CA of odontogenic origin, and 19 patients (43%) had CA of a non-odontogenic origin (Fig. 2). There was no significant difference in gender- (*p* = 0.540) or age-distribution (*p* = 0.261) between the two groups (Table 1). The overall 1-year mortality rate was 3/44 patients (7%). In all patients, attempts during admission were made to identify the underlying infectious focus. In the non-odontogenic group, sinusitis was the most likely source of infection (6/19, 32%). Patients with type 2 diabetes (T2D) were solely represented in the odontogenic group (*p* = 0.014), and in six of seven T2D patients, the hemoglobin A_1c_ (HbA1c) level was obtained on admission. Five of those patients had HbA1c levels above 7.7 mmol/L with a mean of 10.6 mmol/L ± 2.5 mmol/L. Immunocompromising factors were present in five patients of the non-odontogenic group (*p* = 0.011) and included patients with intravenous substance abuse, malignant melanoma, multiple myeloma, untreated HIV, and one patient receiving infliximab for Crohn’s disease. Predisposing risk factors were present in four patients in the odontogenic group: One patient had neurosurgery performed 98 days prior to hospitalization due to the removal of suprasellar meningioma, one patient had experienced a minor head trauma 29 days prior to hospitalization, one patient had a patent foramen ovale, and one patient had sinus-surgery performed 16 days and 9 days prior to hospitalization due to acute odontogenic sinusitis.

**Table 1:** Patient characteristics

Characteristics	Total, *n* (%)	CA with non-odontogenic origin, *n* (%)	CA with odontogenic origin, *n* (%)
Patients	44 (100)	19 (100)	25 (100)
Age, years, mean ± SD	61	60 ± 11.9	54 ± 20.6
Gender, male	28 (64)	11 (58)	17 (68)
Likely source of CA			
Unknown	1	1	0
Odontogenic infection	25	0	25
Sinusitis	6	6	1^a^
Ear infection	2	2	0
Other contiguous infections	3	3	0
Bacteremic spread of systemic infection	4	4	0
Other	3	3	0
Comorbidities and immunocompromising factors	16 (36)^b^	8 (42)^c^	8 (32)^c^
T2D	7 (16)	0	7 (28)
Alcohol abuse	6 (14)	4 (21)	2 (8)
IV substance abuse	1 (2)	1 (5)	0
Organ transplant	0	0	0
Solid cancer	1 (2)	1 (5)	0
Haematological cancer	1 (2)	1 (5)	0
Untreated HIV	1 (2)	1 (5)	0
Other immunosuppressive therapy	1 (2)	1 (5)	0
Predisposing risk factors	16 (36)^d^	7 (37)^d^	4 (16)
Neurosurgery/ENT surgery	6 (14)	4 (21)	2 (8)
Head trauma	2 (5)	1 (5)	1 (4)
Predisposing heart condition	2 (5)	1 (5)	1 (4)
Liver cirrhosis	1 (2)	1 (5)	0
Cranial defect	1 (2)	1 (5)	0
Mortality			
< 28-day mortality	3 (7)	3 (16)	0
> 28-day mortality	4 (9)	2 (11)	2 (8)

Abbreviations: *CA*, cerebral abscess; *SD*, standard deviation; *T2D*, type two diabetes; *IV*, intravenous; *HIV*, human immunodeficiency virus; *ENT*, ear, nose, and throat

^a^1 patient had unilateral odontogenic sinusitis

^b^2 patients had more than one comorbidity

^c^1 patient had more than one comorbidity

^d^1 patient had more than one predisposing risk factor

### Radiographic and clinical examinations

PRs were available for radiographic examination in 28 patients (64%), and in eight patients (18%), CT scans of the maxillary and mandibular regions were examined. The remaining eight patients did not have available radiographic material. The four examiners were in complete agreement in 149/168 observations (89%) from the PR examinations. Statistical measures of the intra-rater reliabilities were computed with a mean *κ*-value at 0.89 ± 0.05 (range 0.86 to 0.96), and inter-rater reliabilities on the presence of oral pathologic conditions had a mean *κ*-value at 0.86 ± 0.14 (range 0.73 to 1.00) (Supplementary S2). The level of agreement on the presence of oral pathologic conditions was interpreted as substantial and almost perfect.

All 25 patients characterized as CA with odontogenic origin had radiographic material evaluated by the examiners, of which 21 evaluations were based on PRs and four on CT scans of the maxillary and mandibular region. The examiners agreed on the presence of radiographic pathologic conditions in all 25 patients. Oral pathologic conditions, as assessed radiographically by the examiners, are shown in Table 2. Fourteen patients with CA of odontogenic origin (56%) also had a clinical dental examination conducted during hospitalization by an oral and maxillofacial surgeon. In these patients, apical periodontitis was recorded in 12 patients, severe periodontitis in five patients, and pericoronitis in one patient. Tooth extractions were performed in 12/14 patients during hospitalization (Supplementary S3). Multiple oral pathologic conditions were present in 18 out of the 25 patients (72%), and 22 patients (88%) had radiographic and/or clinical signs of either apical periodontitis or pericoronitis leaving three patients (12%) with severe periodontitis only. The oral pathologic conditions involved molars in 19 patients (76%), and there was no predilection for mandibular versus maxillary involvement. One patient had a registered contiguous spread of infection due to unilateral odontogenic sinusitis with periapical pathology in the ipsilateral maxillary region.

**Table 2:** Oral pathologic conditions in patients characterized as CA with odontogenic origin

Radiographic oral pathology	*n* (%)
Apical radiolucency	18 (72)
Severe marginal bone loss	14 (56)
Pericoronal radiolucency	5 (20)

Of the 19 patients characterized as CA with non-odontogenic origin, seven had a PR evaluated, and four had a CT scan evaluated. Radiographic material of the maxillary and mandibular region was not available in eight patients. Radiographic pathologic conditions were present in 6/11 possible patients, where five patients had apical radiolucencies, six had severe bone loss, and one patient had pericoronal radiolucency.

### Microbiology

**Fig. 3:**
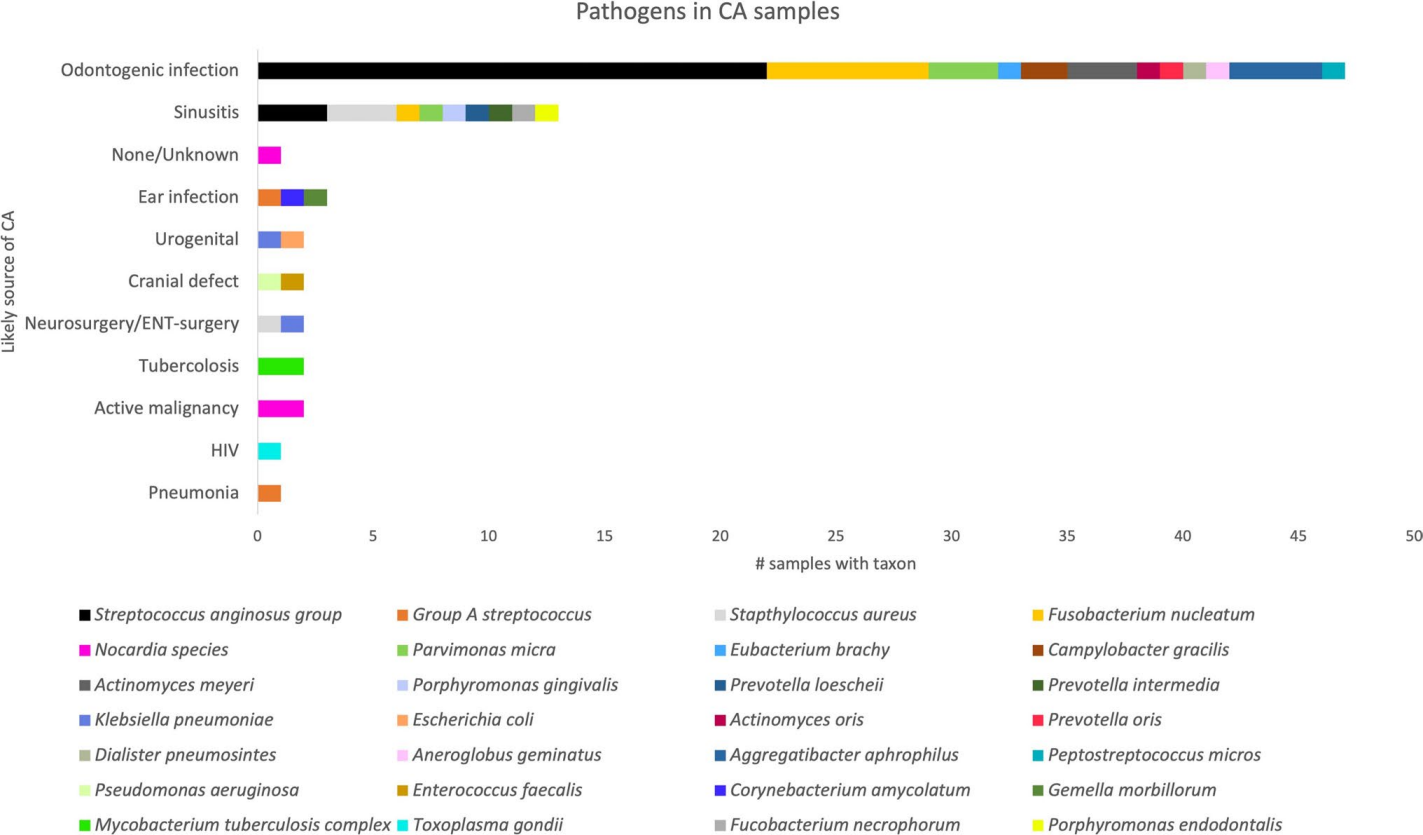
Bacterial spectrum and correlation of pathogens with presumed cause of CA formation

Microorganisms were isolated from pus samples in all 44 patients of which 17 patients (39%) had a confirmed polymicrobial infection. Conventional Sanger sequencing of 16 s rDNA was performed on 21 samples (48%), whereas 23 samples (52%) were solely cultured. Six patients (14%) had both sequencing and culturing performed. Seventeen patients (39%) received preoperative antibiotics for a median duration of 2 days, including eight patients (18%) with a registered monomicrobial infection. Multiple microorganisms were isolated, among which gram-positive bacteria accounted for the majority. Bacteria from the *Streptococcus anginosus* group (SAG) were present in 25/44 patients (57%) with 16 patients (36%) infected with unspecified subspecies and nine patients (20%) infected with *S. intermedius*. The patients characterized with odontogenic CA had bacteria isolated from either the SAG (22/25, 88%), *Fusobacterium nucleatum* (7/25, 28%), *Aggregatibacter aphrophilus* (4/25, 16%), or a combination of these. In 8/25 patients (32%), additional oral bacteria were isolated, and 12/25 patients (48%) had a confirmed polymicrobial infection. All microorganisms involved in the odontogenic group are listed (Supplementary S3). Microorganisms that are a part of the microbiota of the oral cavity were exclusively isolated from patients with CA developed from odontogenic infections or sinusitis (Fig. 3). Bacteria isolated from the SAG were more common in CA patients with odontogenic origin (*p* < 0.01). The remaining patients in the non-odontogenic group had a wide variety of pathogens isolated, including three cases of cerebral nocardiosis, two cases of cerebral tuberculomas, and one patient with cerebral toxoplasmosis (Fig. 3).

## Discussion

In the present study, 57% of the CA patients were characterized as having an odontogenic origin. This proportion is higher than previously reported [1, 3, 5, 6]. The thorough examination of radiographic material revealed oral pathologic conditions in 11 patients (25%) during hospitalization, in whom predisposing infections were initially not registered. Several other studies have reported a substantial group of patients with CA of unknown origin [1, 2]; however, only one patient had an unknown origin in this study. This finding suggests that some patients in previous studies categorized with unknown origin may have had an overlooked odontogenic predisposing infection. As oral pathologic conditions do not always exhibit any clinical signs or symptoms [12], more attention to the clinical dental and radiographic examination during hospitalization is recommended.

The oral pathologic conditions observed in the odontogenic CA group are similar to those reported in previous studies [11, 13]. They include signs of apical periodontitis and severe periodontitis involving molar teeth with no predilection for mandibular versus maxillary teeth. Also, signs of pericoronitis were present in several patients. Most patients had signs of multiple oral pathologic conditions indicating that neglect of oral care could be a potential issue in some of these patients. The identified oral pathologic conditions were all inflammatory diseases that are expected to increase the risk and load of bacteremia, leading to the dissemination of oral microorganisms causing systemic and distant site infections [14–16]. Dental procedures performed before admission were not systematically registered in the medical journals and were therefore not included for further analysis in this study. However, a recent study found no increased risk of developing CA after invasive dental procedures [17]. Eleven patients characterized as CA with odontogenic origin had solely oral pathologic conditions revealed on PRs, and these were not examined during hospitalization by an oral and maxillofacial surgeon. Although PRs compared with other radiographic techniques have a limited value detecting some oral pathologic conditions [12, 18], a high agreement among the examiners in this study validated the actual presence of oral pathologic conditions on admission in these 11 patients. In the non-odontogenic group, 8 patients did not have radiographic material of the mandibular and maxillary region, as an obvious infectious focus was found at an early stage during hospitalization. In both groups, four patients did not have an independent radiographic examination, as CT-material were evaluated in cooperation between the examiners. The evaluation of CT scans was done without the examiners having access to background information on the patients; however, it is a limit to the radiographic evaluation.

Sinusitis was the second most common predisposing infection, and attention should be paid to the fact that maxillary sinusitis may be odontogenic as well. Odontogenic sinusitis is a prevalent and underdiagnosed disease caused by apical periodontitis or iatrogenic extrusion of foreign bodies into the maxillary sinuses and is strongly associated with unilateral maxillary sinusitis [19, 20]. Therefore, CA patients with unilateral maxillary sinusitis should have an oral examination conducted as odontogenic sinusitis usually requires combined dental and medical treatment [21].

In four patients characterized as having odontogenic CA, predisposing risk factors were present. In three out of four patients, the predisposing risk factors were related to prior surgical interventions or head trauma. However, the pathogens isolated were microorganisms predominantly found in the oral cavity and the risk factors were not attributed to CA formation in the medical records. One of the patients received sinus surgery prior to hospitalization due to oral pathologic conditions, and one patient had a patent foramen ovale, which is not per se the reason for CA formation. Therefore, all four patients were evaluated to be justifiable and characterized as odontogenic CA.

Diabetes has previously been suggested as a risk factor that may exacerbate odontogenic infections [22], and T2D with poor glycemic control is well known to have a bidirectional relationship with periodontal disease, due to negative effects on the immune system [23]. Based on the findings from the present study, diabetic patients should pay extra attention to preserve healthy oral conditions, as T2D patients were overrepresented among patients with odontogenic CA (*p* = 0.014). Also, in 5/6 patients, the HbA1c level was above 7.7 mmol/L indicating that poor glycemic control may be correlated with odontogenic CA formation.

Microorganisms from the SAG were the most prevalent pathogens isolated in the present cohort. The SAG consists of *Streptococci constellatus*, *Streptococci intermedius*, and *Streptococci anginosus*, and the group is frequently commensals of the oral cavity, but also isolated from the upper respiratory, gastrointestinal, and genitourinary tracts [24]. These bacteria are well-recognized for causing central nervous system infections, and in recent decades, the incidence of CAs with SAG bacteria has increased [25]. They are often isolated from odontogenic infectious lesions including periapical abscesses, periodontal abscesses, and pericoronitis [26–30]. Also, *S. constellatus* and *S. intermedius* found in dental plaque have been associated with the development of periodontal disease [31]. A recent study from Sweden concluded that *S. anginosus* alone, as a single species, accounted for over 20% of oral bacterial infections [30], and a previous study showed a very close similarity between brain and oral isolates of SAG in a CA patient with periodontitis, where the use of highly discriminative phenotypic and genetic fingerprint techniques could not exclude that microorganisms involved in CA formation came from the periodontal pocket [32]. However, it is important to bear in mind that microorganisms from the SAG, especially *S. anginosus*, are commonly isolated from gastrointestinal and genitourinary tract infections as well [24]. Though, none of the patients in the odontogenic group had any signs of infections in addition to the oral cavity.

The SAG bacteria use several strategies to invade and colonize host cells and tissues. However, the understanding of their virulence factors and especially their regulation is limited [33]. In biofilms, the regulation of virulence genes is controlled through quorum sensing [34], and it has been suggested that synergy between SAG bacteria and oral anaerobes results in enhanced growth of SAG bacteria [28].

The analyses of the relationship between the suspected reason of CA development and the microbiological composition of the brain pus indicate that when bacteria from the SAG or other oral bacteria are isolated in the brain pus, a thorough dental examination should be conducted. This corresponds well with previous findings, where isolation of SAG from brain pus has led to the classification of the CA as odontogenic origin [35, 36]. While several odontogenic CA patients in the present cohort had a monomicrobial infection of SAG isolates or other oral microorganisms (Supplementary S3), other studies suggest that odontogenic CAs are predominantly associated with polymicrobial infections [2]. However, as the oral cavity harboring > 700 bacterial species of which only 68% have been cultured [24], culture-based methods cannot be expected to detect all slow-growing and fastidious oral microorganisms. Additionally, culturing may be impaired by the administration of antibiotics preoperatively and by the nutritional media used for sample transportation and general conditions of transportation [37]. In polymicrobial samples, conventional 16 s rDNA sanger sequencing is also inadequate to discriminate among all taxa, and some samples may produce false-negative results due to low bacteria concentration [37]. Recent studies using metagenomic and next-generation 16 s rDNA sequencing methods disclosed a wide variety of oral microorganisms previously unknown to be involved in CA development, and a larger polymicrobial proportion than previously thought was found [35–37]. Therefore, a monomicrobial isolate should be interpreted with caution and should not rule out a potential odontogenic origin.

In the non-odontogenic group, 6/11 patients had radiographic pathologic conditions. This finding is not surprising, as there is a high prevalence of asymptomatic oral infections in the general population [38, 39]. It is presently unknown why a limited proportion of these conditions develop into CA. Future investigations should focus on the host-pathogen interaction, as an aberrant immune response to bacterial infections in some patients may allow CA development. The identification of any immune deficiency in combination with specific odontogenic infections may assist in developing preventive measures to reduce the risk of developing CA.

This study has several limitations. The main limitation is the risk of ascertainment bias as there could be an unavoidable risk of favoring the characterization of patients with odontogenic origin. However, the risk was kept at a minimum due to the evaluation of intra- and inter-rater reliability during the radiographic assessment and the use of pre-defined criteria when characterizing CA patients with odontogenic origin. Even though CA patients were documented in the DASGIB database, some patients were referred from other hospitals whereby clinical information from hospital admission in certain cases were limited. Due to the limited focus on oral health during the hospitalization, the main recordings of these parameters were based on radiographic information, on registrations from the oral maxillofacial surgeon that evaluated a selected proportion of the patients, and on occasional remarks in the medical records. The cohort examined is well-defined, and the strength of this study includes the continuous registration of data in a predefined registration form in the DASGIB database.

However, when using the three criteria to characterize odontogenic CA patients, there is a potential risk to overestimate the possible frequency of CA with odontogenic origin, as microorganisms normally present in the oral cavity might be involved in infections external to the oral cavity.

## Conclusions

The observations from the present retrospective cohort study show an association between CA with oral microorganisms and the presence of oral pathological conditions, where no other infectious foci in the body could be found. Even though strict criteria were applied to characterize odontogenic CA, the frequency of odontogenic CA in this study should be interpreted with caution, as there is a risk of overestimating the frequency of odontogenic CA due to undiagnosed infections. However, when oral microorganisms were isolated from the brain pus, CA patients were likely to have an odontogenic or sinusitis-predisposing infection. A thorough radiographic and dental examination should be conducted during hospitalization in these patients. Patients with T2D should be advised to collaborate closely with their general dental practitioner to stay free of infectious odontogenic foci.

## Supplementary information

Below is the link to the electronic supplementary material.

Supplementary file1 (DOCX 33 KB)

## Data Availability

Raw data is not available, but analyzed data is available in supplementary.
