# Supplementary material for: Cerebral abscesses with odontogenic origin: a population-based cohort study
Source: Clin Oral Investig. 2023 Mar 31;27(7):3639–48. doi: 10.1007/s00784-023-04976-6 (PMC10329578; doi:10.1007/s00784-023-04976-6)
Supplement: Supplementary file 1 — Supplementary file1 (DOCX 33 KB) [file 784_2023_4976_MOESM1_ESM.docx]

# Supplementary

**Supplementary Table 1: Radiographic assessment**

| Deep radiolucent lesions in the dentin  Radiolucency in the inner 2/3 of the dentin  Root remnants without restorations |
| --- |
| Apical radiolucency  Apical bone destruction noticeably larger than a widened periodontal space |
| Endodontic treated teeth  Pulp cavities or root canals containing radiopaque material |
| Retained teeth  All teeth covered with bone  Partially or fully impacted teeth |
| Severe marginal bone loss indicating periodontitis  Patients had severe periodontitis registered if <66% remaining bone were present |
| Pericoronal radiolucency  Patients with radiographic radiolucency of 2.5mm or larger in any dimension related to the third molars were registered |

Examiners: Frederik V. B. Jespersen (FVBJ), Signe U.-B. Hansen (SUBH), Simon S. Jensen (SSJ), and Merete Markvart (MM)

**Supplementary Table 2: Inter-rater reliability kappa values**

| Apical radiolucency | Rater 1 | Rater 2 | Rater 3 | Rater 4 |
| --- | --- | --- | --- | --- |
| Rater 1 | NA | NA | NA | NA |
| Rater 2 | 0.811 | NA | NA | NA |
| Rater 3 | 0.9 | 0.909 | NA | NA |
| Rater 4 | 0.9 | 0.727 | 0.81 | NA |
| Overall agreement^a^ | 0.84 | | | |
| Pericoronal radiolucency | Rater 1 | Rater 2 | Rater 3 | Rater 4 |
| Rater 1 | NA | NA | NA | NA |
| Rater 2 | 1 | NA | NA | NA |
| Rater 3 | 1 | 1 | NA | NA |
| Rater 4 | 1 | 1 | 1 | NA |
| Overall agreement^a^ | 1 | | | |
| Severe marginal bone loss | Rater 1 | Rater 2 | Rater 3 | Rater 4 |
| Rater 1 | NA | NA | NA | NA |
| Rater 2 | 0.924 | NA | NA | NA |
| Rater 3 | 0.924 | 0.85 | NA | NA |
| Rater 4 | 0.529 | 0.59 | 0.59 | NA |
| Overall agreement^a^ | 0.73 | | | |
| Retained teeth | Rater 1 | Rater 2 | Rater 3 | Rater 4 |
| Rater 1 | NA | NA | NA | NA |
| Rater 2 | 0.759 | NA | NA | NA |
| Rater 3 | 1 | 0.759 | NA | NA |
| Rater 4 | 0.868 | 0.887 | 0.868 | NA |
| Overall agreement^a^ | 0.86 | | | |
| Endodontic treated teeth | Rater 1 | Rater 2 | Rater 3 | Rater 4 |
| Rater 1 | NA | NA | NA | NA |
| Rater 2 | 1 | NA | NA | NA |
| Rater 3 | 1 | 1 | NA | NA |
| Rater 4 | 1 | 1 | 1 | NA |
| Overall agreement^a^ | 1 | | | |
| Deep radiolucent lesions in the dentin | Rater 1 | Rater 2 | Rater 3 | Rater 4 |
| Rater 1 | NA | NA | NA | NA |
| Rater 2 | 0.639 | NA | NA | NA |
| Rater 3 | 0.858 | 0.505 | NA | NA |
| Rater 4 | 0.929 | 0.571 | 0.929 | NA |
| Overall agreement^a^ | 0.74 | | | |

^a^Kappa was computed for each coder pair then averaged to provide a single index of inter-rater reliability (Light, 1971).

**Supplementary Table 3: Comorbidities, risk factors,** **microbiology, oral pathologic conditions, and dental treatment during hospitalization in odontogenic CA patients**

| Sex, age | Comorbidities and immunocompromising factors | Risk factors | Microbiology^a^ | Clinical or radiographic odontogenic pathology (Tooth no.)^b^ | Dental treatment (Tooth no.) |
| --- | --- | --- | --- | --- | --- |
| F, 73 | None | None | *Streptococcus intermedius* (S)  *S. anginosus gr.* (C) | Apical periodontitis (36) | Extraction (36) |
| M, 52 | T2D | None | *Streptococcus intermedius* (S)  *Fusobacterium nucleatum* (S)  *S. anginosus gr.* (C) | Pericoronitis (48) | Extraction (48,47) |
| M, 62 | None | None | *Streptococcus intermedius* (S)  *Fusobacterium nucleatum* (S)  *Parvimonas micra* (S)  *Eubacterium brachy* (S)  *Campylobacter gracilis* (S)  *Actinomyces meyeri* (S)  *S. anginosus gr.* (C) | Pericoronal radiolucency (38);  Severe bone loss (18,28) | None |
| M, 62 | None | None | *Streptococcus intermedius* (S)  *Actinomyces oris (C)* | Severe generalized bone loss | None |
| M, 54 | T2D  Alcohol abuse | None | *Fusobacterium nucleatum* (S)  *Parvimonas micra* (S)  *Prevotella oris* (S)  *Dialister pneumosintes* (S)  *Aneroglobus geminatus* (S) | Apical periodontitis (17,16,25,26);  Severe generalized periodontits | Extraction (17,16,25,26,27,28,37,38,48,47) |
| M, 21 | None | None | *S. anginosus gr.* (C) | Pericoronal radiolucency (38) | None |
| M, 49 | None | None | *Fusobacterium nucleatum (C)* | Apical periodontitis (13,21,22,32,42);  Severe bone loss (33) | Extraction (13,22,32,33, 42) |
| M, 64 | Alcohol abuse | None | *S. anginosus gr.* (C) | Apical periodontitis (18,17,16,26);  Severe generalized periodontitis | Extraction (18,17,16,15,14,11,25,26) |
| M, 64 | T2D | None | *S. anginosus gr.* (C) | Apical periodontitis (35,45) | Extraction (35,45) |
| F, 55 | None | Neurosurgery | *S. anginosus gr.* (C) | Apical periodontitis (31,32, 43) | Extraction (31,43) |
| M, 48 | None | None | *S. anginosus gr.* (C)  *Aggregatibacter aphrophilus (C)* | Apical radiolucency (16) | None |
| F, 51 | None | Patent foramen ovale | *S. anginosus gr.* (S)  *Peptostreptococcus micros* (S) | Apical radiolucency (45) | None |
| F, 74 | None | None | *S. anginosus gr.* (C) | Apical periodontitis (18,36,37);  Severe generalized bone loss | Extraction (18,36,37) |
| M, 45 | None | None | *S. anginosus gr.* (C)  *Aggregatibacter aphrophilus (C)* | Apical peridontitis (36);  Periocoronal radiolucency (38,48) | Extraction (38) |
| M, 75 | None | None | *S. anginosus gr.* (C)  *Campylobacter gracilis* (C) | Apical periodontits (35) | Extraction (35) |
| M, 67 | None | None | *Parvimonas micra* (S)  *S. anginosus gr.* (C) | Apical periodontitis (47,46);  Severe periodontitis (17,16,26) | None |
| M, 59 | T2D | None | *S. anginosus gr.* (C) | Apical periodontitis (15,23,24);  Severe generalized bone loss | Extraction (15,23,24) |
| F, 63 | None | None | *Streptococcus intermedius* (S)  *Fusobacterium nucleatum* (S) | Apical radiolucency (36,46) | None |
| M, 61 | T2D | None | *Aggregatibacter aphrophilus* (C) | Pericoronal radiolucency (38) | None |
| F, 78 | None | Head trauma | *S. anginosus gr.* (C) | Severe periodontitis (26) | None |
| F, 61 | T2D | None | *S. anginosus gr.* (C) | Apical radiolucency (21);  Severe bone loss (16) | None |
| M, 71 | None | None | *S. anginosus gr.* (C) | Severe generalized bone loss | None |
| M, 61 | None | None | *S. anginosus gr.* (C)  *Aggregatibacter aphrophilus* (C)  *Fusobacterium nucleatum* (C)  *Actinomyces meyeri* (C) | Apical periodontitis (46);  Severe generalized periodontitis | Extraction (36,47,46) |
| F, 66 | None | Odontogenic sinusitis | *Streptococcus intermedius* (S) | Apical radiolucency (16,25,27,33,36,43);  Severe generalized bone loss | None |
| M, 64 | T2D | None | *Streptococcus intermedius* (C)  *Fusobacterium nucleatum* (C)  *Actinomyces meyeri* (C) | Apical radiolucency (25);  Severe generalized bone loss | None |

^a^ S = 16S rDNA sequenzing, C = Unspecified culturing

^b^All clinical diagnoses were registered by the oral and maxillofacial surgeon and all radiographic pathologic conditions were registered by the examiners. Only the clinical diagnosis is mentioned if similar clinical and radiographic conditions were registered.
